# Effect of Preoperative Antiplatelet Therapy with Acetylsalicylic Acid on Complications and Recurrence in Patients Requiring Drainage of Chronic Subdural Hematomas: a Systematic Review and Meta-analysis

**DOI:** 10.1007/s00701-025-06605-5

**Published:** 2025-07-25

**Authors:** Mariana Agudelo-Arrieta, Felipe Marin-Navas, Angie Carolina Alonso-Ramirez, Alex Taub-Krivoy, Julian Alfonso Sierra-Peña, Oscar Hernando Feo-Lee, Miguel Enrique Berbeo-Calderon

**Affiliations:** 1 Faculty of Medicine, Pontificia Universidad Javeriana, Bogotá, Colombia; 2 Department of Neurosurgery, Hospital Universitario San Ignacio, Bogotá, Colombia; 3 Department of Neurology, Stroke Division, Beth Israel Deaconess Medical Center and Harvard Medical School, Boston, MA USA

**Keywords:** Antiplatelet therapy, Chronic subdural hematoma, Neovascularization, Risk factors

## Abstract

**Objective:**

To assess the effect of preoperative acetylsalicylic acid use on the risk of recurrence and complications in patients undergoing surgical drainage of chronic subdural hematoma (cSDH) in light of the widespread use of acetylsalicylic acid for cardiovascular prevention among the growing elderly population.

**Methods:**

A systematic review and meta-analysis were conducted in accordance with PRISMA 2020 guidelines. A comprehensive search was performed on May 31st, 2025, across Pubmed, EMBASE, LILACS, and Web of Science. Eligible studies included cohort studies and randomized controlled trials (RCT) involving patients with cSDH undergoing surgical drainage who were on preoperative acetylsalicylic acid therapy. Outcomes analyzed included mortality, morbidity, perioperative and postoperative complications, and recurrence rates. The risk of bias was assessed using the Newcastle–Ottawa Scale (NOS) for cohort studies and the RoB 2 tool for RCT. Meta-analysis was conducted using a random-effects model, and heterogeneity was evaluated using the I^2^ statistics.

**Results:**

The systematic review included eight cohort studies (seven retrospective, one prospective) and one RCT, comprising a total of 3,209 participants, of whom 1,152 (35.8%) had received acetylsalicylic acid preoperatively. Our meta-analysis of 1556 observations found no significant difference in recurrence risk between acetylsalicylic acid users and non-users [RR: 0.99; 95% CI: 0.68—1.45; p = 0.96; I^2^ = 11.2%]. However, preoperatively acetylsalicylic acid use was associated with a significantly increased risk of thromboembolic events [RR: 2.89; 95% CI: 1.18—7.11; p = 0.02; I^2^ = 0.0%].

**Conclusion:**

Limited evidence suggests that preoperative acetylsalicylic acid use does not significantly increase the risk of cSDH recurrence in patients undergoing surgical drainage. Conversely, these patients may be at increased risk of thromboembolic events. This highlights the importance of careful patient selection and individualized management. Further research is warranted to guide clinical decision-making regarding acetylsalicylic acid therapy in this population.

**Supplementary information:**

The online version contains supplementary material available at 10.1007/s00701-025-06605-5.

## Introduction

Chronic subdural hematoma (cSDH) is a prevalent neurosurgical condition, particularly among the older population, characterized by the insidious accumulation of blood within the subdural space, frequently following minor or unnoticed head trauma [4, 6, 9, 30]. Its global incidence is rising parallel to population aging, rendering it an increasingly significant public health issue [5, 9, 12, 15].

A major clinical challenge emerges when patients receiving chronic acetylsalicylic acid therapy require surgical evacuation of a cSDH [6, 10, 30]. In such cases, clinicians are confronted with the need to balance the potential for increased risk of hematoma recurrence or perioperative hemorrhage associated with continued antiplatelet use against the heightened risk of thromboembolic complications if acetylsalicylic acid is discontinued [12]. Despite the clinical importance of this dilemma, the current body of evidence remains inconclusive, primarily based on retrospective studies with heterogeneous designs and variable methodological quality, thereby limiting the formulation of evidence-based management guidelines [3, 6, 8, 10, 12, 16].

In light of these complexities, there exists a pressing need to synthesize the available evidence regarding the impact of preoperative acetylsalicylic acid therapy on both the risk of cSDH recurrence and the incidence of thromboembolic events following surgical intervention. The present systematic review and meta-analysis address this critical knowledge gap by evaluating data from randomized controlled trials (RCTs) and observational studies. These findings aim to inform clinical decision-making, guide perioperative management strategies, and delineate priorities for future research.

## Methods

This study followed the Preferred Reporting Items for Systematic Reviews and Meta-Analyses (PRISMA) guidelines. The protocol was registered in the International Prospective Register of Systematic Reviews (PROSPERO) under the registration number CRD42024613444.

### Criteria For Considering Studies

#### Study types

We conducted a systematic review of relevant studies published in English or Spanish without restrictions on publication date. Eligible studies were either RCTs or observational studies, including cohort and case–control studies, that meet the following criteria: 1) studies including patients with cSDH requiring surgical drainage; 2) studies including patients receiving acetylsalicylic acid therapy for either primary or secondary prevention of cardiovascular events; and (3) studies reporting of at least one relevant clinical outcome, including mortality, morbidity, perioperative or postoperative complications, or recurrence rates. The following publication types were excluded: case reports, case series, dissertations, book chapters, study protocols, narrative or systematic reviews, conference abstracts, letters to the editor, editorials, and commentaries.

#### Types of participants

This study included female and male participants over ≥ 18 years old with cSDH requiring surgical drainage. Pediatric population (under 18 years of age) and patients with acute subdural hematomas were excluded.

#### Types of interventions

To be eligible for inclusion in this study, articles were required to evaluate the effect of acetylsalicylic acid therapy on recurrence and complications in cases of cSDH requiring surgical drainage. The intervention was limited exclusively to oral administration of acetylsalicylic acid. Only studies that reported outcome data specifically for patients receiving acetylsalicylic acid were included. Studies in which acetylsalicylic acid was grouped with other antiplatelet agents—such as clopidogrel or ticagrelor—without disaggregated data were excluded to prevent confounding and maintain the specificity of the analysis.

#### Outcomes

The primary outcomes analyzed were recurrence and thromboembolic events. Secondary outcomes described narratively included mortality, morbidity, and peri- or postoperative complications. Studies with incomplete data and irrelevant outcomes have been excluded. Recurrence was defined as radiological evidence of increased cSDH volume at or near the surgical site, accompanied by clinical symptoms requiring reoperation within six months of the initial surgery.

#### Searching methods

Our search on May 31 st, 2025 encompassed Pubmed, EMBASE, LILACS, and Web of Science using relevant MESH terms and free-text terms such as “chronic subdural hematoma” AND “acetylsalicylic acid” OR “aspirin”.

#### Selection of studies

In the initial phase, titles and abstracts were independently screened by two reviewers, who selected studies for inclusion based on the aforementioned eligibility criteria. The screening process was facilitated by using the web-based tool Rayyan-QCRI, which streamlined the review workflow by efficiently identifying relevant studies and removing duplicate records [23]. Discrepancies in study selection were resolved through consensus or, when necessary, by consultation with a third reviewer. Subsequently, full-text articles were independently evaluated by a different pair of reviewers to determine eligibility in accordance with the established inclusion criteria. Any disagreements arising during this stage were addressed through discussion and resolved by consensus or third-party adjudication.

#### Assessment of the risk of bias in included studies

The evaluation of data was conducted in accordance with the criteria outlined in the *Cochrane Handbook for Systematic Reviews of Interventions*. To assess the methodological quality of the included studies, the Newcastle–Ottawa Scale (NOS) was employed for the appraisal of non-randomized clinical studies [28]. RCTs were evaluated using the Cochrane Risk of Bias 2.0 (RoB 2) tool [17]. This assessment was performed independently by two reviewers, who determined the risk of bias for each study based on the explicit criteria and guidance provided by the respective tools. In cases of disagreement, consensus was reached through consultation with a third reviewer, who was blinded to the initial evaluations.

#### Statistical analysis

Baseline demographics and study characteristics were summarized using descriptive statistics. A meta-analysis of binary outcomes was performed to compare the risk of cSDH recurrence and thromboembolic events between acetylsalicylic acid users and non-users. Effect sizes were reported as relative risks (RR) with 95% confidence intervals (CI). A random-effects model was applied to account for heterogeneity among the studies. Heterogeneity was assessed using the I^2^ statistic, with values ≥ 50% indicating substantial heterogeneity and ≥ 75% indicating considerable heterogeneity. P-values < 0.05 were considered statistically significant. Publication bias was assessed using funnel plot analyses to visualize the distribution of effect sizes. When possible, subgroup analyses were conducted based on the study design and antiplatelet exposure to explore their potential impact on the overall results. All analyses and data visualizations were performed using R software, version 2024.04.0 + 735 [25].

## Results

### Study selection and quality assessment

**Fig. 1:**
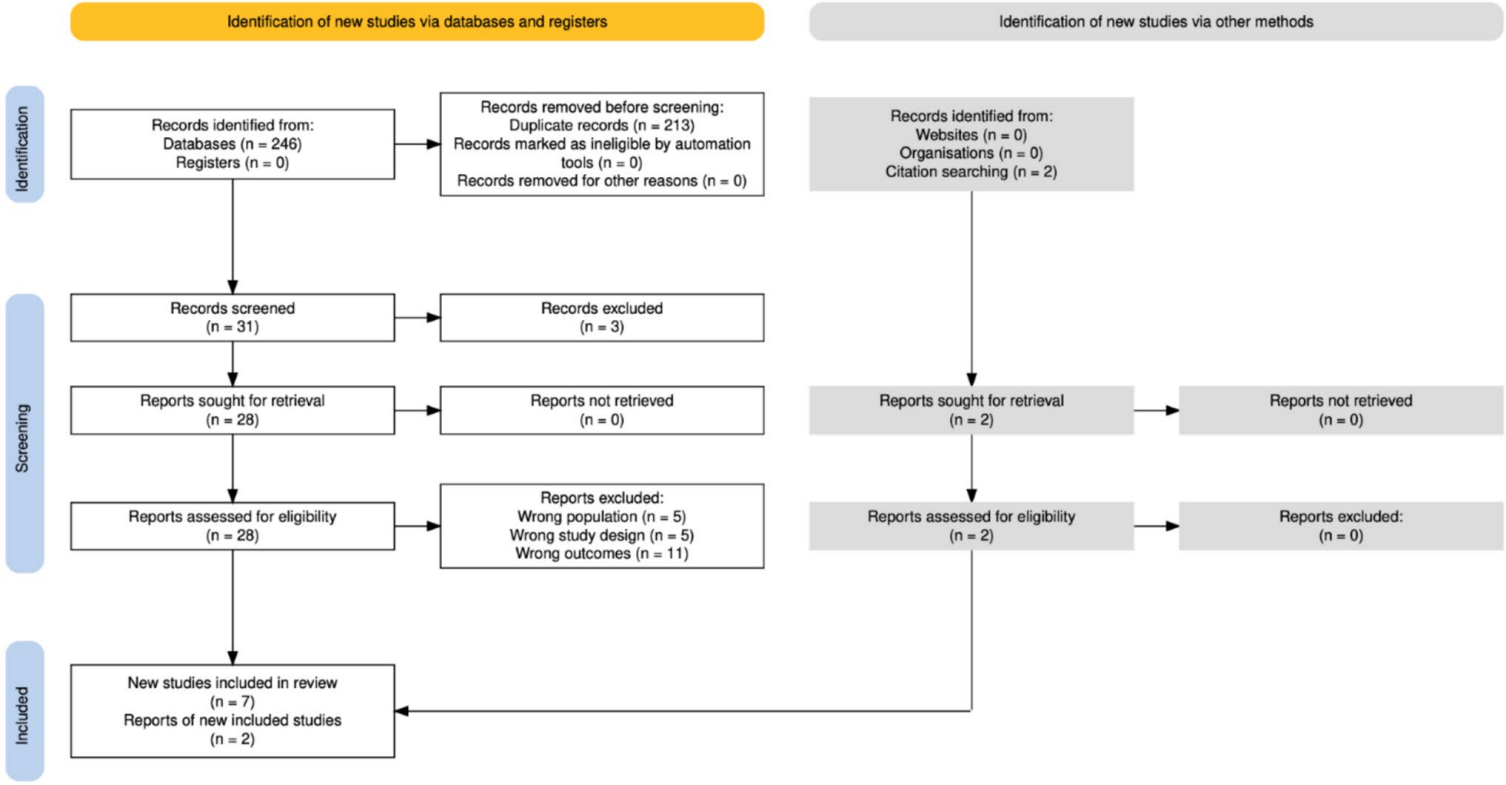
PRISMA Flow Diagram

The initial database search yielded a total of 246 records. After removing 213 duplicate entries, 31 unique records remained for screening. Titles and abstracts of these 31 records were reviewed for relevance. Three records were excluded based on predefined exclusion criteria, including language restrictions, classification as conference abstracts, and the absence of relevant outcomes. This resulted in 28 records deemed eligible for full-text review. The full-text articles of these records were assessed for eligibility. Upon detailed assessment of the full-text articles, 21 studies were excluded due to non-relevant outcomes or incomplete data. A revision of the references within the selected articles led to the identification of two additional studies that met the inclusion criteria. Finally, a total of 9 studies were deemed eligible and were included in the systematic review. These studies were evaluated in detail to extract relevant data and assess the risk of bias [7, 11, 12, 20, 21, 24, 26]. The study selection process is summarized in the PRISMA flow diagram **(**Fig. 1**)**.

Flow diagram illustrating the study selection process for the systematic review.

The methodological quality of the included studies was assessed using the NOS for cohort studies; Table 1 summarizes the quality of each evaluated study (Table 1). The quality assessment revealed a range in methodological rigor among the included studies, with total scores ranging from 5 to 8. Three studies were rated as"fair quality"with scores of 5 and 6, mainly due to limitations in the representativeness of the exposed cohort, comparability, and length of follow-up. In contrast, five studies were rated as"good quality"with scores of 7 and 8, indicating strong cohort representativeness, confounder control, and sufficient follow-up (Table 1). These findings suggest that most included studies possess adequate methodological quality, ensuring reliable and interpretable results for the systematic review and meta-analysis. However, the identified limitations in some studies underscore the need for cautious interpretation of the overall conclusions.

**Table 1:**
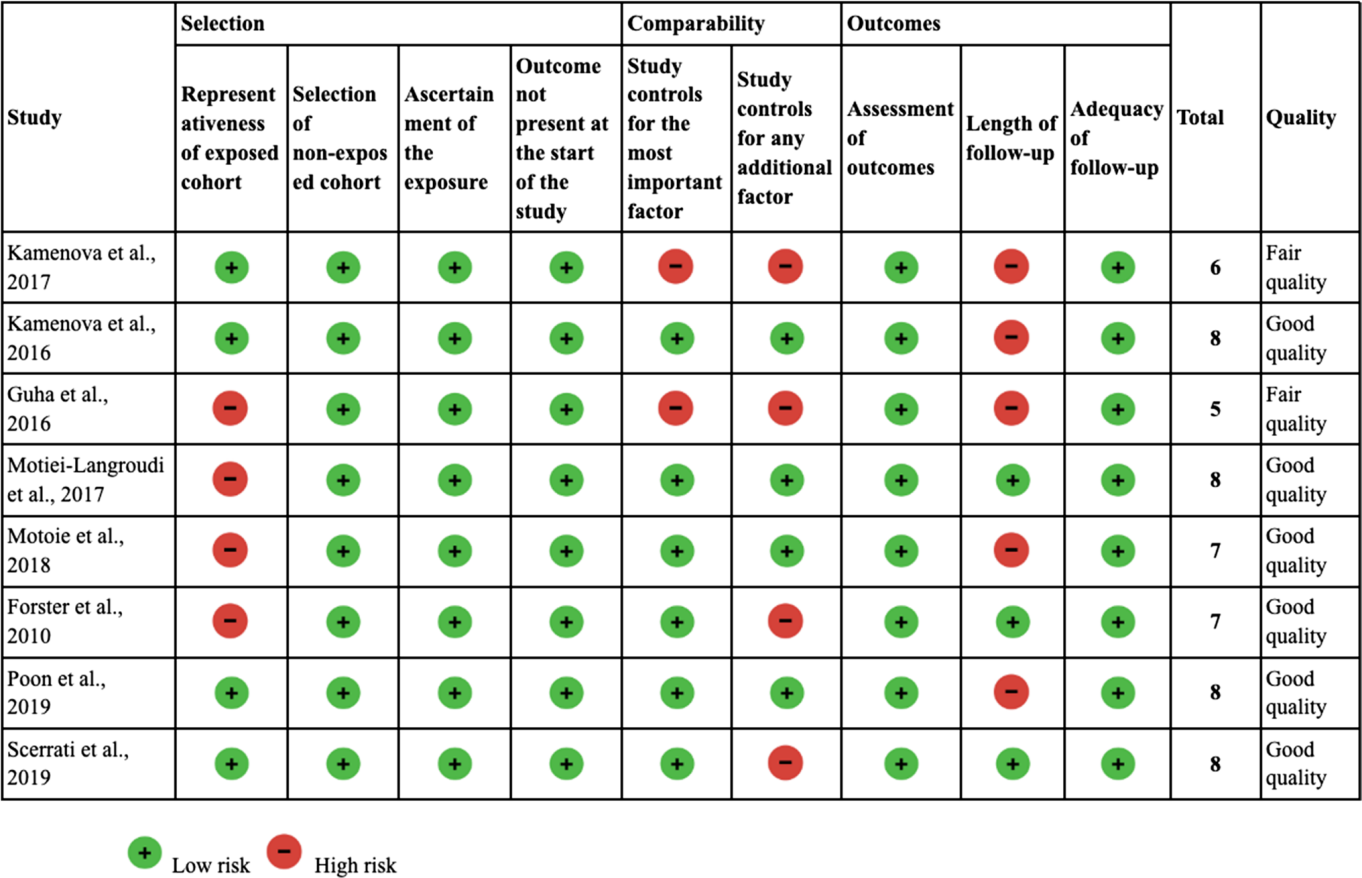
Newcastle-Ottawa Scale for Cohort Studies

The risk of bias assessment for the included RCT by Kamenova et al. 2025 was performed using the Cochrane Risk of Bias 2 (RoB 2) tool [14]. The evaluation indicated a low risk of bias across most domains, including the randomization process, deviations from intended interventions, and outcome measurement. However, some concerns were noted in the domain of missing outcome data due to incomplete follow-up, which could potentially impact the internal validity of the study. Overall, the study demonstrated a good methodological quality (Table 2).

**Table 2:**
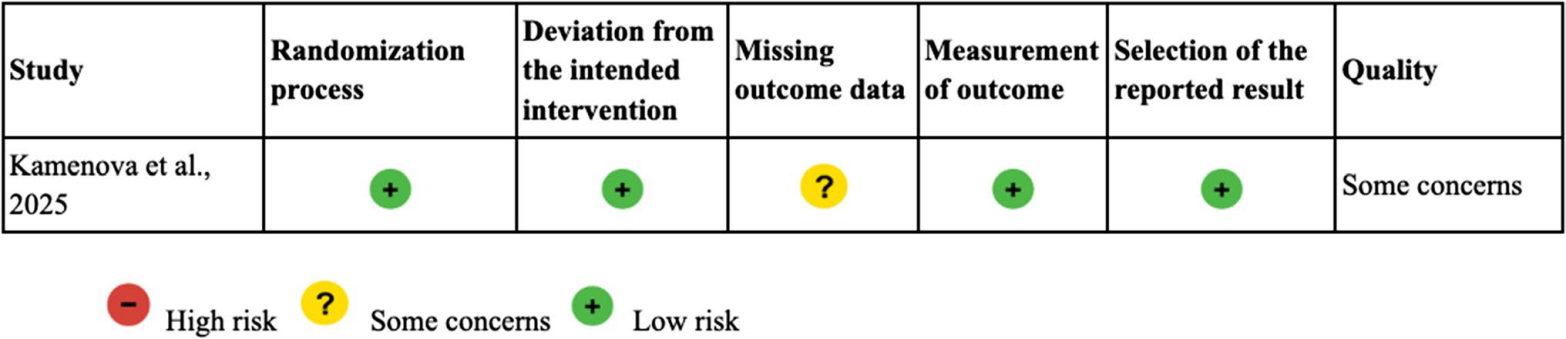
RoB2 for Randomised Trials

### Characteristics of Included Studies

The systematic review included a total of seven retrospective cohort studies [7, 11, 12, 20, 21, 26], one prospective cohort study [24], and one RCT [14], comprising a combined sample of 3,209 participants diagnosed with cSDH. These studies specifically investigated the effect of preoperative antiplatelet therapy with acetylsalicylic acid on postoperative complications and mortality in patients undergoing drainage procedures (3,030 participants). The study populations primarily consisted of adults and elderly individuals who required surgical intervention for cSDH. Sample sizes ranged from 140 to 817 participants per study.

While two articles did not specify subdural hematoma sides [24, 26], a similar distribution was noted among those that did: 13.9% left, 12.9% right, and 13.4% bilateral. The most prevalent surgical technique employed was the burr hole method (87.5%), with subdurostomy used in 79.6% of cases **(**Table 3). Several critical areas needed more comprehensive data, including the Glasgow Coma Scale upon admission, the etiology of SDH, time to surgical management, and the status of neurological impairment pre-surgery.

**Table 3:** Study Designs and General Characteristics

Study, Year	Country	Design	Patients included	Patients undergoing surgery	Surgical technique	Use of acetylsalicylic acid before surgery (%)
Foster et al., 2010 [7]	Germany	Retrospective	144	144	Burr hole craniostomy or craniotomy	38 (26.39)
Kamenova et al., 2016 [13]	Switzerland	Retrospective	140	140	Burr hole craniostomy	140 (100)
Guha et al., 2016 [11]	Canada and Ireland	Retrospective	479	479	Burr hole craniostomy or craniotomy	136 (28.39)
Kamenova et al., 2017 [12]	Switzerland	Retrospective	198	198	Burr hole craniostomy	198 (100)
Motiei-Langroudi et al., 2017 [20]	USA	Retrospective	325	446*	Burr hole craniostomy or craniotomy	81 (24.9)
Motoie et al., 2018 [21]	Japan	Retrospective	787	787	Burr hole craniostomy	98 (12.45)
Poon et al., 2019 [24]	United Kingdom	Prospective	817	770	Burr hole craniostomy	142 (17.38)
Scerrati et al., 2019 [26]	Italy	Retrospective	164	164	No information	164 (100)
Kamenova et al., 2025 [14]	Switzerland	RCT	155	155	Double Burr hole craniostomy	155 (100)

*In cases of bilateral hematomas, each side was analyzed separately as an individual case

*RCT*; Randomized controlled trial

Of the 3,209 patients included in the study, 1,152 (35.8%) had used acetylsalicylic acid before surgery. Four studies [7, 13, 22, 26] reported the number of patients who consumed this medication on the day of surgery, accounting for 206 patients.

Most studies did not report the average time of discontinuation of acetylsalicylic acid before the procedure. Kamenova et al., reported that 80 patients (74.1%) took acetylsalicylic acid on the day of the procedure, 12 patients (11.1%) suspended the medication one day before the procedure, 8 patients (7.4%) two days before, 3 patients (2.8%) three days before, 5 patients (4.6%) four days before, and 32 patients (22.9%) discontinued acetylsalicylic acid at least five days before the procedure [13]. Although the average acetylsalicylic acid suspension time was not reported, five authors did provide data on the number of patients who did not discontinue acetylsalicylic acid, as well as those who suspended it less than 5 days and more than 5 days before surgery [7, 12, 13, 26]. Among those who reported an acetylsalicylic acid suspension group, this subgroup represented approximately 50% of the studied population. Furthermore, in studies that subdivided patients into those who discontinued acetylsalicylic acid more or less than 5 days before surgery, each subgroup accounted for approximately one-quarter of the total cohort [13, 14, 21]. Notably, only Kamenova et al. 2017 stratified patients based on a 7-day threshold, with one group suspending acetylsalicylic acid for fewer than 7 days and a"control"group with suspension exceeding 7 days [12]. Unfortunately, none of these studies specified outcomes such as intraoperative bleeding, recurrence, or the need for reoperation within these specific suspension subgroups, thereby limiting the ability to perform a detailed analysis of clinical outcomes. (Table 4**).**

**Table 4:** Protocols Used to Suspend Acetylsalicylic Acid Before Surgery

Author	Year	Patients without Acetylsalicylic acid suspension (%)	Acetylsalicylic acid suspended < 5 days before surgery (%)	Acetylsalicylic acid suspended > 5 days before surgery (%)
Forster et al. [7]	2010	12 (31)	26 (68)	0
Kamenova et al. [13]	2016	80 (57)	28 (20)	32 (23)
Kamenova et al. [12]	2017	0	26 (Referred to as the Acetylsalicylic acid group in the article) * (13)	172 (Referred to as the control group in the article) * (87)
Scerrati et al. [26]	2019	69 (42)	59 (36)	36 (22)
Kamenova et al. [14]	2025	78 (50)	71 (45)	6 (5)

* The cut-off point is 7 days

The included studies offered limited information regarding the indications for acetylsalicylic acid therapy, patient risk profiles, and the necessity for antiplatelet reversal. Most studies identified recurrence and reoperation as primary outcomes; however, none sought to establish specific target rates. Instead, the rates of these outcomes were compared between patients who continued acetylsalicylic acid during the initial intervention and those in whom acetylsalicylic acid was suspended. Across all studies, the reported rates were consistent with those previously described in the literature—approximately 9–13%—with no statistically significant differences observed between the two groups.

### Perioperative and postoperative management in patients with cSDH

Regarding surgical specifics, there was insufficient information on the timing of surgeries between groups using and not using acetylsalicylic acid, intraoperative blood loss, and hemorrhagic complications. Complications such as infection and mortality were reported in only three studies [12, 14, 25], with no clear differentiation observed between patients using acetylsalicylic acid and those who did not.

Concerning acetylsalicylic acid post-surgical management, only four authors specified the average time (in days) before acetylsalicylic acid was resumed in patients whose use was suspended upon admission. [11, 12, 24]. Among these, two studies reported a similar resumption time of 52 days [11, 12], while two others categorized resumption times into different intervals ranging from the first to the twelfth week [13, 24], without comparison to a group in which acetylsalicylic acid was not suspended. None of the studies reported specific bleeding in other organs or systems. Two authors reported the resumption of pharmacological thromboprophylaxis with low molecular weight heparin: Kamenova et al. within 24–48 h and Guha et al. within 48–72 h post-drainage [11, 12]. Information on hospitalization duration, morbidity, and modified Rankin Scale scores was poorly reported in the analyzed studies.

Only four authors reported clinical follow-up data [11, 12] with a mean duration of 90.7 days. Imaging follow-up was inconsistently reported, with an average duration of 65.9 days (38–180 days). Due to the limited number of comparative studies and the heterogeneity and incompleteness of the available data, a meta-analysis could not be conducted for any of the outcomes described in this section. Consequently, the results are presented qualitatively.

### Cardiovascular events following cSDH drainage in patients with prior acetylsalicylic acid use

Only three studies reported cardiovascular events (acute myocardial infarction with or without ST elevation, angina, vascular stroke, or arterial ischemia): 11 acute myocardial infarctions, 1 femoral artery occlusion, and 6 unspecified events. All of these events occurred in patients who had used acetylsalicylic acid before surgery [11, 12, 14]. Of the 11 patients with acute myocardial infarction, 6 were in the group where acetylsalicylic acid was discontinued during the postoperative period, while 3 continued taking it. For two patients, this information was not reported. The femoral artery occlusion occurred in a patient who had discontinued the drug. Because two of the three studies reporting these events were single-arm cohorts that did not include patients without prior acetylsalicylic acid use, a quantitative analysis could not be performed.

### Thromboembolic events following cSDH drainage in patients with prior acetylsalicylic acid use

A total of 37 thromboembolic events, including pulmonary embolism (PE) and deep vein thrombosis (DVT), were reported across five studies. One study documented three cases of pulmonary embolism in patients who continued acetylsalicylic acid postoperatively, one of which resulted in the patient's death [13]. Another study reported 14 thromboembolic events (without distinguishing between PE and DVT), of which three occurred in patients who continued acetylsalicylic acid after the surgery and eleven in patients who had used acetylsalicylic acid preoperatively but discontinued it before the procedure and did not restart it postoperatively [12]. Two additional studies each reported six events: four in patients with prior acetylsalicylic acid use and two in those without previous exposure. One of those studies noted that none of the patients who experienced thromboembolic events and had a history of acetylsalicylic acid use had restarted the medication at the time of the event [11]. Another study reported eight thromboembolic events, equally distributed between acetylsalicylic acid users and non-users [24].

**Fig. 2:**
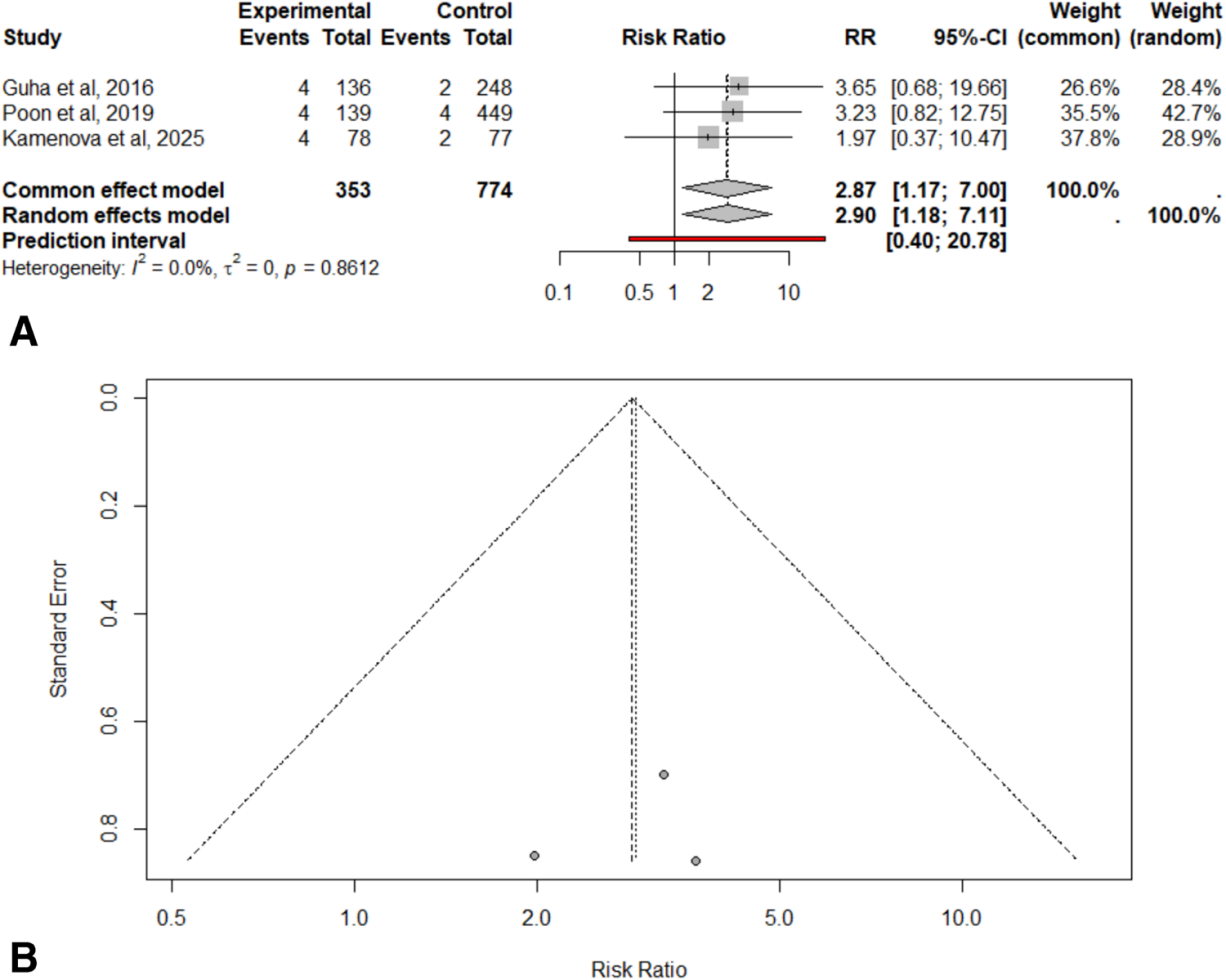
Relative risk (RR) of thromboembolic events in patients with cSDH with prior acetylsalicylic acid use versus those without. **A.** This forest plot illustrates the risk of thromboembolic events in patients with cSDH with prior acetylsalicylic acid use compared to those without. The pooled effect estimate using a random-effects model indicated a RR of 2.90 (95% CI 1.18–7.11), meaning a significant difference in thromboembolic risk between the two groups. The analysis showed low heterogeneity among the studies, with an I2 of 0.0% **B.** Funnel plot detailing publication bias. The figure shows visual symmetry, suggesting no potential publication bias

The pooled RR of thromboembolic events risk in patients with cSDH was 2.8995 (95% CI: 1.1823—7.1112), indicating a statistically significant difference in thromboembolic risk between acetylsalicylic acid users and non-users (z = 2.33, p = 0.0200). This pooled estimate was derived from only three studies that included acetylsalicylic acid users and non-users, as the remaining studies were single-arm cohorts, including patients with prior acetylsalicylic acid use. The prediction interval ranged from 0.40 to 20.78, highlighting the considerable uncertainty in individual study effects. Heterogeneity analysis revealed low heterogeneity across studies, with an I^2^ value of 0.0% (Fig. 2A). Given the low heterogeneity observed, sensitivity analyses were not conducted. The funnel plot showed no visual asymmetry, suggesting no publication bias (Fig. 2B). Subgroup and sensitivity analyses were not carried out due to low heterogeneity.

### Recurrence risk following cSDH drainage in patients with prior acetylsalicylic acid use

Eight studies reported data on the recurrence of cSDH in relation to acetylsalicylic acid use. Across these studies, recurrence was observed in acetylsalicylic acid users and non-users, with mixed findings. One study reported 19 recurrences (13.6%) among 140 acetylsalicylic acid users. In contrast, another reported 18 recurrences (16.7%) among 108 patients who resumed acetylsalicylic acid postoperatively, compared to only one recurrence (3.1%) among 32 patients who did not resume the medication. In a separate study, 24 recurrences were documented, of which 3 occurred in the acetylsalicylic acid group and 21 in the control group. Another study noted that 60.6% of patients requiring reoperation had taken acetylsalicylic acid. Based on event counts alone, recurrence was documented in 12 and 11 acetylsalicylic acid users in two studies, compared to 22 and 85 recurrences in non-users, respectively. Additional studies reported 10 recurrences in acetylsalicylic acid users versus 66 in non-users, 20 in acetylsalicylic acid users with no data from a control group, and 8 versus 7 recurrences in acetylsalicylic acid users and non-users, respectively.

**Fig. 3:**
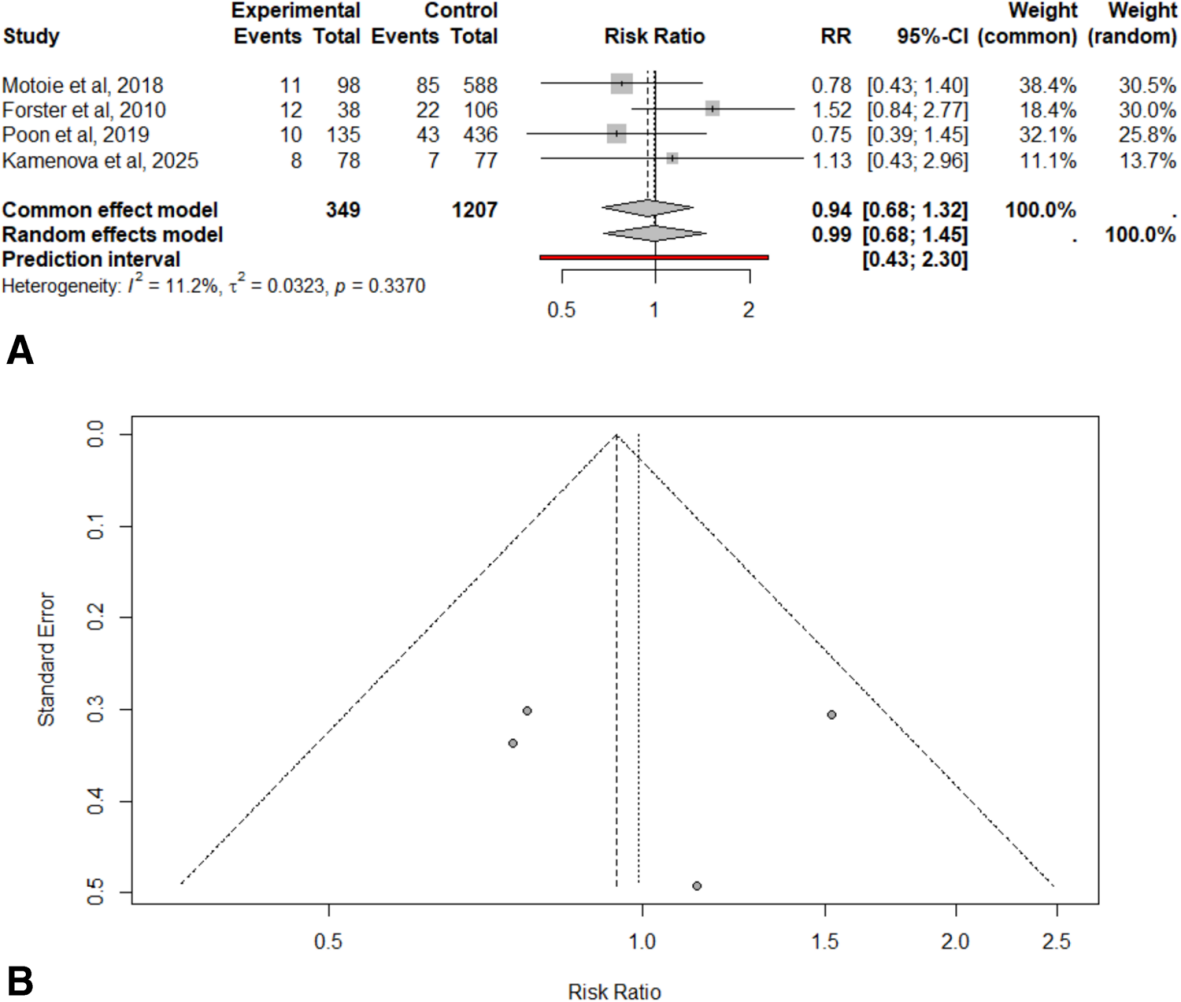
Relative risk (RR) of cSDH recurrence in patients with cSDH with prior acetylsalicylic acid use versus those without. **A.** This forest plot illustrates the risk of recurrent cSDH comparing patients with prior acetylsalicylic acid use to those without. The pooled effect estimate using a random-effects model indicated a RR of 0.99 (95% CI 0.68–1.46), meaning no significant difference in recurrence risk between the two groups. The analysis showed low heterogeneity among the studies, with an I2 of 11.2%. **B.** Funnel plot detailing publication bias. The figure shows visual symmetry, suggesting no potential publication bias

The pooled RR of recurrence of cSDH was 0.9914 (95% CI: 0.6780–1.4497), indicating no significant difference in recurrence risk between acetylsalicylic acid users and non-users (z = –0.04, p = 0.9644). This estimate was based on four studies that included both acetylsalicylic acid users and non-users; the remaining studies were single-arm cohorts that only included patients with prior acetylsalicylic acid use and were therefore excluded from the meta-analysis. Notably, two of the included studies may have featured a contaminated case group, as they did not clearly distinguish between patients receiving acetylsalicylic acid monotherapy and those on dual antiplatelet therapy (DAPT), making these groups not mutually exclusive. To explore the potential influence of this overlap, a subgroup analysis was conducted to assess whether the presence of DAPT may have affected the overall findings. Heterogeneity was low, with an I^2^ value of 11.2% (Fig. 3A), and thus sensitivity analyses were not performed. Visual inspection of the funnel plot revealed no asymmetry, suggesting no evidence of publication bias (Fig. 3B).

### Subgroup Analysis and meta-regression

Subgroup analyses within the random effects model revealed no statistically significant differences in the effect of preoperative acetylsalicylic acid use on cSDH recurrence when stratified by study design (retrospective cohort, prospective cohort, and RCT; Q = 0.76, p = 0.6828) or by type of antiplatelet exposure (acetylsalicylic acid only vs. possible dual antiplatelet therapy; Q = 0.30, p = 0.5855). Although the point estimates varied slightly between subgroups, particularly in antiplatelet exposure, the RR estimates were broadly comparable, and the wide CI combined with the small number of included studies suggest limited statistical power to detect meaningful differences (Supplementary Figs. 1A and 1B).

Meta-regression showed no significant effect of either antiplatelet exposure type (QM(1) = 0.24, p = 0.62) or study design (QM(2) = 0.43, p = 0.81) on the risk of recurrence. In both models, between-study heterogeneity remained largely unexplained (R^2^ = 0%), and the CI for regression coefficients was wide, reflecting limited statistical power.

## Discussion

cSDH represents a prevalent neurosurgical condition, particularly affecting older people, with rising incidence and significant functional and economic impact [9, 18, 22, 29, 30]. The widespread use of antiplatelet and anticoagulant therapies within this population complicates perioperative management, given the inherent balancing act between the risks of hemorrhage and thromboembolic events [5, 6, 12, 15, 30]. Although acetylsalicylic acid is widely prescribed for cardiovascular prevention, its perioperative safety in cSDH patients remains controversial, and clinical practice varies widely [1, 19, 31].

Our systematic review and meta-analysis provides updated insights into the perioperative management of patients with cSDH receiving acetylsalicylic acid therapy. In the context of an aging population with increasing rates of both cSDH and cardiovascular comorbidities, determining whether to continue or suspend acetylsalicylic acid before surgery remains a daily clinical challenge. The studies included in our analysis focused primarily on adult and elderly patients, reflecting real-world practice. Despite rigorous inclusion criteria, limitations related to study design, reporting, and heterogeneity must be acknowledged when interpreting the results.

Our meta-analysis found no significant difference in the recurrence risk of cSDH between acetylsalicylic acid users and non-users (RR: 0.99; 95% CI: 0.68–1.45), with low heterogeneity (I^2^ = 11.2%). These findings align with those of Poon et al. and Zhang et al., who also reported no significant increase in recurrence among patients receiving antiplatelet therapy [24, 31]. Poon et al. found similar crude recurrence rates across antithrombotic, antiplatelet, and non-antithrombotic groups and emphasized the absence of clear clinical guidelines for managing these medications perioperatively [24]. Zhang et al., in turn, found no statistically significant difference in recurrence among antiplatelet users [31].

In contrast, Wang et al. and Brannigan et al. identified an elevated risk of recurrence associated with both antiplatelet and anticoagulant therapies. Their broader meta-analyses encompassed multiple drug classes, which may account for the discrepancy relative to our acetylsalicylic acid-specific analysis. These observations underscore the necessity for further investigation into whether distinct antithrombotic agents confer differential risks in the context of cSDH recurrence [2, 27].

Despite the overall consistency, significant variability exists in the timing of acetylsalicylic acid suspension across studies, ranging from continued use to several days of preoperative discontinuation. This heterogeneity reflects the lack of standardized protocols and limits the ability to draw definitive conclusions. Moreover, cardiovascular and thromboembolic events were predominantly reported in patients with prior acetylsalicylic acid use, underscoring the need for individualized perioperative decision-making.

Our analysis also identified a significantly higher risk of thromboembolic events in acetylsalicylic acid users (RR = 2.89; 95% CI: 1.18–7.11), echoing the findings of Brannigan et al., who suggested that this association may reflect a combination of baseline cardiovascular comorbidities, perioperative interruption of therapy, and the prothrombotic state associated with surgery [2]. Nevertheless, these findings should be interpreted cautiously due to the limited number of studies and the lack of individual-level data.

Notably, our review included the only available RCT [14], which compared acetylsalicylic acid continuation versus discontinuation. While recurrence rates did not differ significantly, the study reported a lower incidence of cardiovascular events in patients who continued acetylsalicylic acid. These results support the hypothesis that the continuation of acetylsalicylic acid may be safe in selected patients, reinforcing the need to balance hemorrhagic and thrombotic risks on a case-by-case basis.

These results underscore the need for a multidisciplinary approach involving neurosurgeons, neurologists, and cardiologists to optimize perioperative management. Rather than a uniform protocol, patient selection should be guided by clinical context, balancing the risk of bleeding against the risk of thromboembolic events.

In summary, our findings suggest that continuing acetylsalicylic acid therapy in carefully selected patients undergoing surgical drainage for cSDH may be safe and might not increase the risk of recurrence. At the same time, the observed association between acetylsalicylic acid withdrawal and thromboembolic events highlights the potential consequences of discontinuation. However, given the limitations of the available evidence, these conclusions should be interpreted with caution. Future studies should standardize outcome definitions and perioperative management protocols to generate more definitive guidance for clinical practice.

## Limitations

This review has several limitations that must be acknowledged. First, most included studies were retrospective and, therefore, subject to inherent biases, including confounding and selection bias. Reporting of key clinical variables was often incomplete, and there was substantial variability in the definitions of outcomes, which limits comparability across studies.

The timing of acetylsalicylic acid discontinuation before surgery and resumption afterward was inconsistently reported and infrequently described in sufficient detail. This clinically relevant factor could not be adequately analyzed and precluded meaningful subgroup or sensitivity analyses. The lack of standardized perioperative protocols complicates the interpretation of both bleeding and thromboembolic risks and limits the generalizability of our findings.

Another limitation of this review concerns the exclusion of studies that reported aggregated outcomes for mixed antiplatelet therapies. This exclusion was a deliberate methodological decision, as the objective was to evaluate the specific safety profile of acetylsalicylic acid. Including data from other agents with distinct pharmacodynamic properties and bleeding risk profiles could have introduced confounding and diminished the specificity of the findings. Nevertheless, this approach may have contributed to a reduced overall sample size.

Finally, although we incorporated data from the SECA trial, the total number of patients included in the meta-analysis remains limited, and several pooled estimates showed wide confidence intervals. This constrains the statistical power of our findings and highlights the need for cautious interpretation of the results.

Future research should prioritize well-designed, prospective studies with standardized perioperative protocols and rigorous outcome definitions. Transparent reporting of acetylsalicylic acid timing, indications for use, and perioperative events will improve comparability across studies and support the development of evidence-based guidelines for acetylsalicylic acid management in patients undergoing surgical treatment for chronic subdural hematomas.

## Conclusions

In conclusion, while our review suggests that preoperative acetylsalicylic acid use does not appear to increase the risk of recurrence in patients with cSDH significantly, it also indicates a potential association with thromboembolic events that may reflect risks related to its discontinuation. These findings must be interpreted cautiously due to the limited number of studies suitable for quantitative synthesis and the high degree of heterogeneity observed. The findings of this review contribute to the ongoing debate and highlight the urgent need for further research to establish evidence-based guidelines for the management of cSDH in patients on antiplatelet therapy. The evidence remains inconclusive, and clinical decisions should continue to be tailored to individual patient profiles, considering comorbidities and bleeding or thrombotic risk. This review highlights significant gaps in the current literature and reinforces the need for well-designed prospective studies to guide the management of antiplatelet therapy in this patient population.

## Supplementary information

Below is the link to the electronic supplementary material.

ESM 1 (DOCX 71.0 KB)

## Data Availability

No datasets were generated or analysed during the current study.
