# Supplementary material for: Effect of Preoperative Antiplatelet Therapy with Acetylsalicylic Acid on Complications and Recurrence in Patients Requiring Drainage of Chronic Subdural Hematomas: a Systematic Review and Meta-analysis
Source: Acta Neurochir (Wien). 2025 Jul 25;167(1):205. doi: 10.1007/s00701-025-06605-5 (PMC12296801; doi:10.1007/s00701-025-06605-5)
Supplement: Supplementary file 1 — (DOCX 71.0 KB) [file 701_2025_6605_MOESM1_ESM.docx]

**SUPPLEMENTARY MATERIAL**

**Supplementary fig. 1A Subgroup analysis by study design**


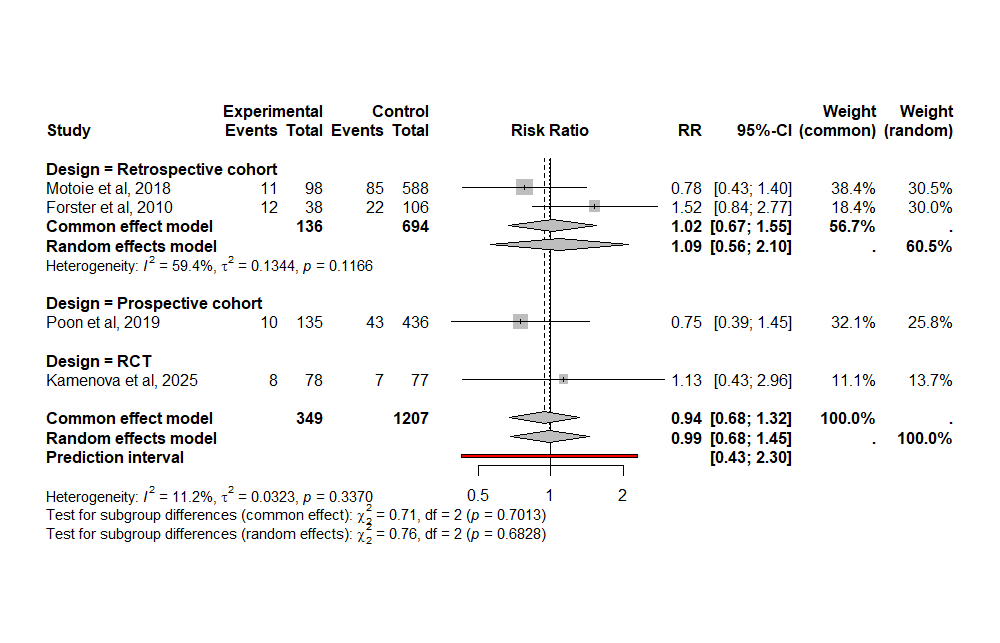


This forest plot displays subgroup analyses based on study design regarding the risk of recurrent cSDH in patients with prior aspirin use compared to those without. The overall pooled effect estimate, using a random-effects model, shows a RR of 0.99 (95% CI 0.68-1.45), indicating no significant difference in recurrence risk. Subgroup analysis reveals consistent findings across study designs, demonstrating low heterogeneity (I2=11.2%)

**Supplementary fig. 1B Subgroup analysis by antiplatelet exposure**


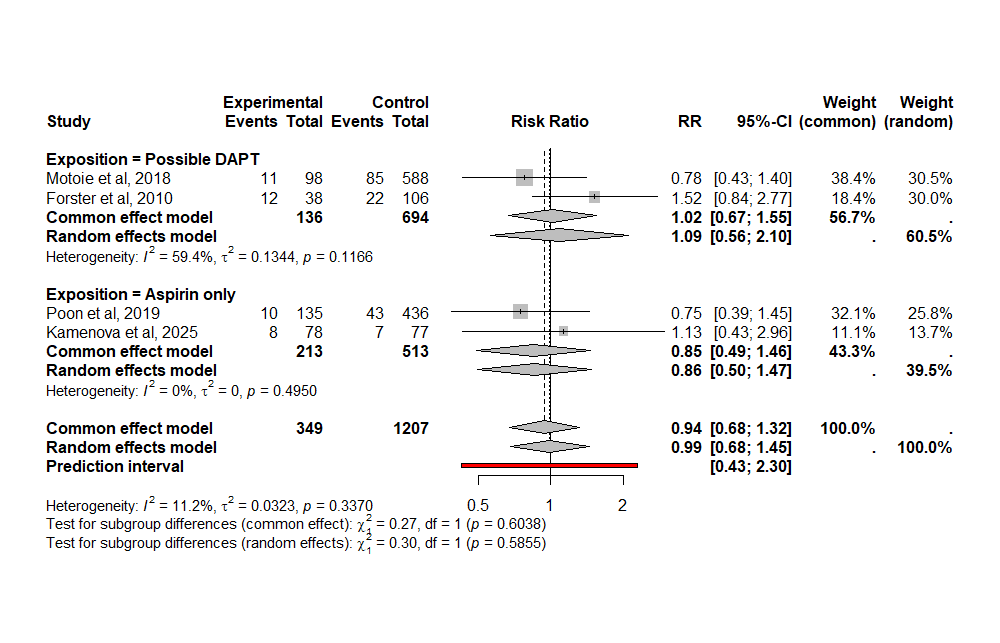


This forest plot displays subgroup analyses based on antiplatelet exposure regarding the risk of recurrent cSDH in patients with prior aspirin use compared to those without. The overall pooled effect estimate, using a random-effects model, shows a RR of 0.99 (95% CI 0.68-1.45), indicating no significant difference in recurrence risk. Subgroup analysis reveals consistent findings across study designs, demonstrating low heterogeneity (I2=11.2%).
